# Supplementary figures and images for: In vitro genome editing activity of Cas9 in somatic cells after random and transposon-based genomic Cas9 integration
Source: PLoS One. 2022 Dec 30;17(12):e0279123. doi: 10.1371/journal.pone.0279123 (PMC9803249; doi:10.1371/journal.pone.0279123)

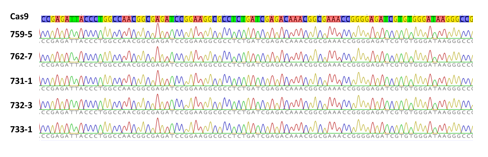

Supplement: S1 Fig — Amplified DNA products can be found in Fig 2(A) and 2(B). (TIF) [file pone.0279123.s001.tif]

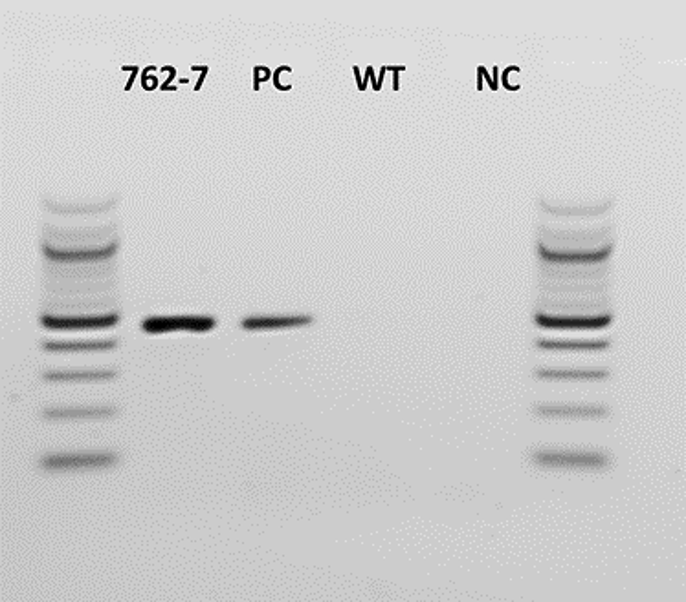

Supplement: S2 Fig — (TIF) [file pone.0279123.s002.tif]

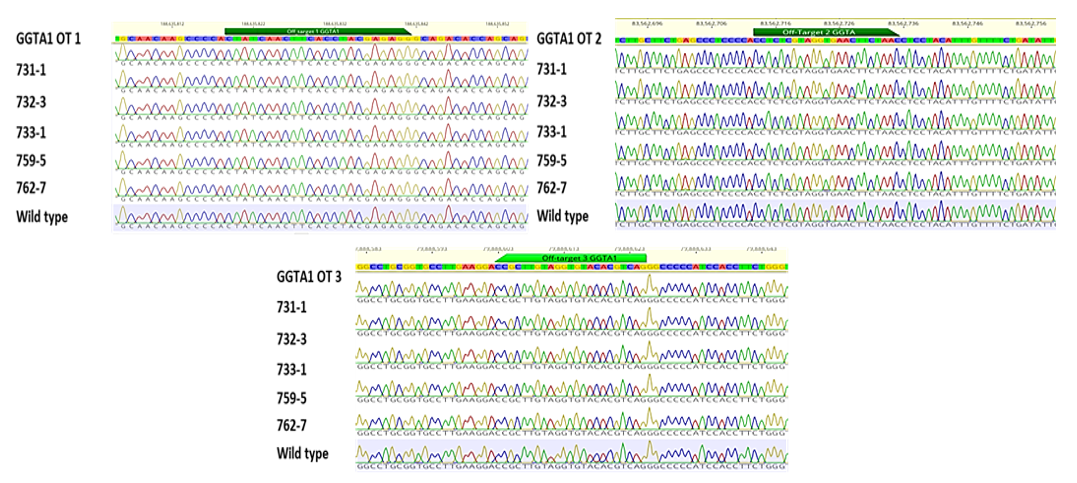

Supplement: S3 Fig — (TIF) [file pone.0279123.s003.tif]

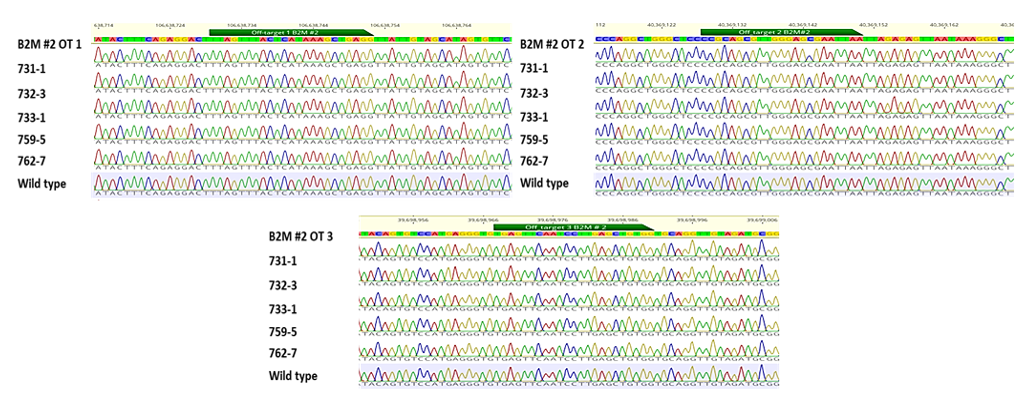

Supplement: S4 Fig — (TIF) [file pone.0279123.s004.tif]

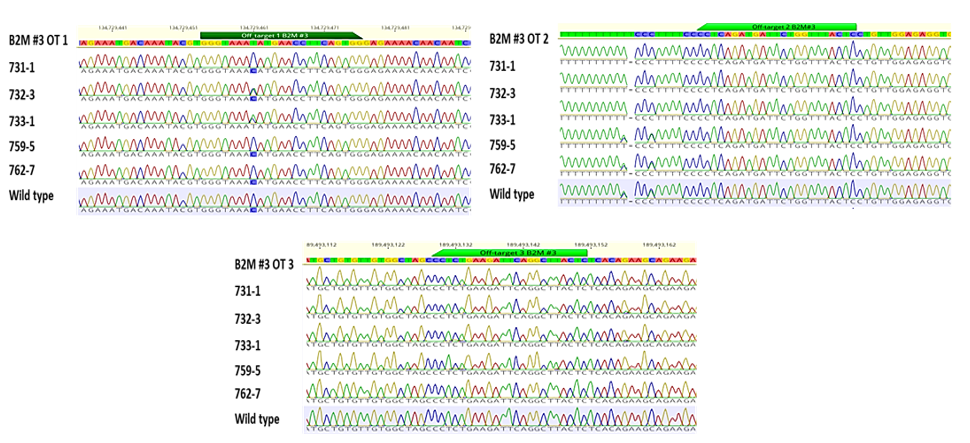

Supplement: S5 Fig — (TIF) [file pone.0279123.s005.tif]

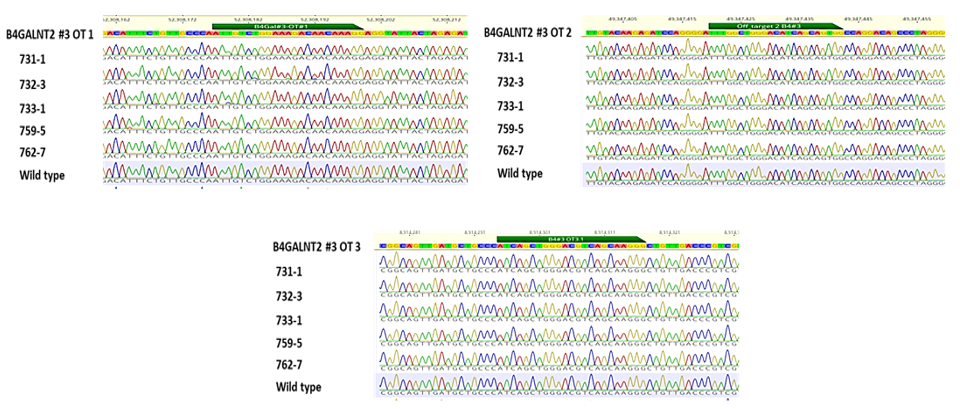

Supplement: S6 Fig — (TIF) [file pone.0279123.s006.tif]

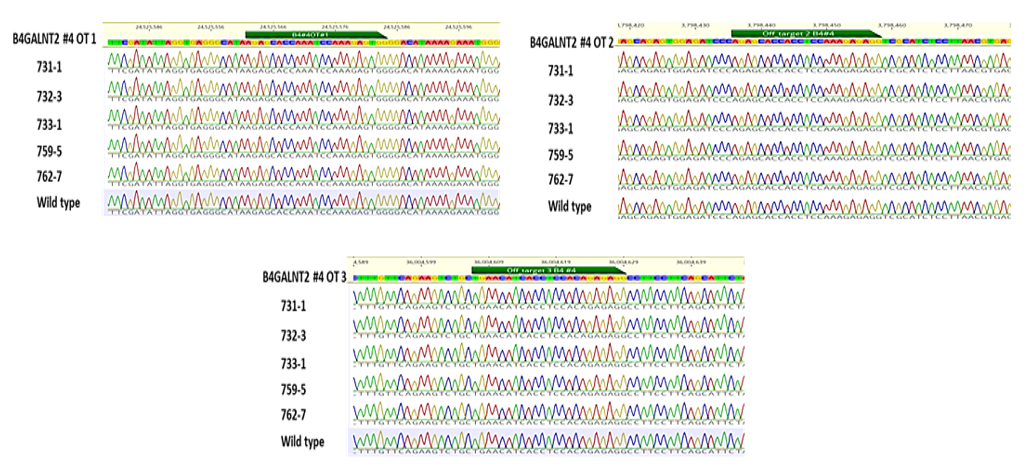

Supplement: S7 Fig — (TIF) [file pone.0279123.s007.tif]
