# Supplementary material for: In vitro genome editing activity of Cas9 in somatic cells after random and transposon-based genomic Cas9 integration
Source: PLoS One. 2022 Dec 30;17(12):e0279123. doi: 10.1371/journal.pone.0279123 (PMC9803249; doi:10.1371/journal.pone.0279123)
Supplement: S1 Table — (DOCX) [file pone.0279123.s008.docx]

| S1 Table. Primers for detecting genome edits. | | | | |
| --- | --- | --- | --- | --- |
| Target | **Primer (5’-3’)** | **Annealing °C** | **Cycles** | **Product** |
| B2M | TGTGGGCAAGTCACTACGTC | 62 | 32 | 763 |
|  | ATGCTCAGATTCGGTTGGCA |  |  |  |
| B4GALNT2 | ACTCTGCATGCCAAGAGTTAAGA | 62 | 35 | 419 |
|  | CCTGGAGACTTTGAGAGCCG |  |  |  |
| GGTA1 | CTAGAAATCCCAGAGGTTAC | 59 | 35 | 553 |
|  | TCCTTGTCCTGGAGGATTCC |  |  |  |
