# Supplementary material for: In vitro genome editing activity of Cas9 in somatic cells after random and transposon-based genomic Cas9 integration
Source: PLoS One. 2022 Dec 30;17(12):e0279123. doi: 10.1371/journal.pone.0279123 (PMC9803249; doi:10.1371/journal.pone.0279123)
Supplement: S2 Table — (DOCX) [file pone.0279123.s009.docx]

| S2 Table. Results from computer assisted sperm morphology for boar 762-7. | | | | | |
| --- | --- | --- | --- | --- | --- |
|  |  | **Fresh Sperm** | | **Frozen Sperm** | |
| Collection | **Animal** | **Mean motility (%)** | **Mean progressive motility (%)** | **Mean motility (%)** | **Mean progressive motility (%)** |
| 1 | 762-7 | 52.9 | 42.5 | NA | NA |
| 2 |  | 54.1 | 16.1 | 5.4 | 2.8 |
| 3 |  | 83.4 | 73.4 | 31 | 27.5 |
