# Supplementary material for: In vitro genome editing activity of Cas9 in somatic cells after random and transposon-based genomic Cas9 integration
Source: PLoS One. 2022 Dec 30;17(12):e0279123. doi: 10.1371/journal.pone.0279123 (PMC9803249; doi:10.1371/journal.pone.0279123)
Supplement: S3 Table — (DOCX) [file pone.0279123.s010.docx]

| S3 Table. Off-target regions. | | | | | |
| --- | --- | --- | --- | --- | --- |
| Guide RNA target | **Off-target region** | **Primer (5’-3’)** | **Annealing °C** | **Cycles** | **Product (bp)** |
| GGTA1 | Chromosome 13  (188,635,819-188,635,841) | CCTAGGCACACAACCTCCAC | 62 | 35 | 311 |
|  |  | AGGTTGCTTACTTCCAGTTCACTT |  |  |  |
|  | Chromosome 3  (83,562,713-83,562,735) | ACCAGAAGGAGGGGAGACTG | 62 | 35 | 452 |
|  |  | GATGGGCCAGAGCTGAAAGTG |  |  |  |
|  | Chromosome 16  (79,888,603-79,888,625) | AGATTCAGCCACAGAAGCCC | 62 | 35 | 194 |
|  |  | CAGGATGAGCTCCACGTCTG |  |  |  |
| B2M #2 | Chromosome 13  (106,638,730-106,638,752) | CTCAGCTTGGGAGCAAAACAC | 62 | 35 | 977 |
|  |  | ACAACACAGGAAGTACAGCCAA |  |  |  |
|  | Chromosome 12  (40,369,128-40,369,150) | GCCAGGGTGTTGCTTTAGGT | 62 | 35 | 858 |
|  |  | TTCCGCTGCAAACACAAACA |  |  |  |
|  | Chromosome 7  (39,698,969-39,698,990) | TAAGGCCACGGAAGTGTGAG | 62 | 35 | 965 |
|  |  | GCCACAGCCCGTCAAATACA |  |  |  |
| B2M #3 | Chromosome 9  (134,729,454-134,729,476) | TCATTGTTGTGGGTCCGTTT | 60 | 35 | 1080 |
|  |  | AGGAATTTTGCAGGTGGTTTG |  |  |  |
|  | Chromosome 5  (59,310,453-59,310,475) | GGAATCGTGCTGAAACGTGG | 62 | 32 | 356 |
|  |  | TGGGAACCAGCAAGGAAAGG |  |  |  |
|  | Chromosome 1  (189,493,129-189,493,151) | TGCGGTTCAGATCCCTTGTT | 62 | 35 | 707 |
|  |  | CCGGATCCTTAACCTGCTTCA |  |  |  |
| B4GALNT2 #3 | Chromosome 7  (49,347,420-49,347,442) | TGCCTTCTGGCTTTGTGGTA | 59 | 32 | 1354 |
|  |  | ATGGGAGAAGAGATGGAGGACA |  |  |  |
|  | Chromosome 9  (8,914,295-8,914,317) | CTAAGATCCCGGTGTTCGGG | 62 | 35 | 930 |
|  |  | GGTCAAGCTCTGGGAACTGG |  |  |  |
|  | Chromosome 11  (52,308,178-52,308,199) | GGGGGACATGTTTTCAGGTT | 59 | 32 | 995 |
|  |  | GCCCTCAGTGTCAATGGTGATA |  |  |  |
| B4GALNT2 #4 | Chromosome 2  (3,798,436-3,798,458) | GATGGTCTCTGCCTAAGCTCC | 62 | 35 | 1089 |
|  |  | GCAATGAAGCTCGGTTCCAG |  |  |  |
|  | Chromosome 9  (36,004,607-36,004,629) | CCTGGTTGTAGCACCCAATGA | 62 | 35 | 671 |
|  |  | CCTGGTTGTAGCACCCAATGA |  |  |  |
|  | Chromosome 17  (24,525,562-24,525,584) | CCCAACGTGATCTGACTCCT | 62 | 32 | 381 |
|  |  | TCCCCCACACAAGGAATTTGT |  |  |  |
