# Supplementary material for: In vitro genome editing activity of Cas9 in somatic cells after random and transposon-based genomic Cas9 integration
Source: PLoS One. 2022 Dec 30;17(12):e0279123. doi: 10.1371/journal.pone.0279123 (PMC9803249; doi:10.1371/journal.pone.0279123)
Supplement: Attachment — Submitted filename: Review Plos One.docx [file pone.0279123.s011.docx]

This manuscript focused on the production of transgenic pig cells and animals by using random and transposon-based integration of a constitutive expression of Cas9 protein for in vitro evaluation. They envision that these animals will serve for the production of various animal models of human disease by in vivo therapy with the sgRNA. Their approach aims to overcome the limitations of the SCNT efficiency and unforeseen abnormalities caused by the same procedure. They also expect that Cas9 expressing animals could be further expanded by breeding.

Major: There is no major objection to this manuscript. The manuscript is overall well written and easy to follow.

Minor: Some sentences could be improved such as:

Line 53. Instead of ‘Humans show a higher anatomical…’, use ‘Pigs show a higher anatomical… resemblance to human than rodents, which still…’. When we talk about animal models, we refer to them as references/models for humans. The way how it is written seems a bit the opposite.

Missing reference in Line 82. ‘In mice, several attempts of in vivo tissue modifications… successful (Reference).’

Some typos also need to be addressed, such as Buffer instead of ‘puffer’; ‘Promoter instead of promotor’; ‘SYBR instead of SYBER’.

Also, some sentence clarifications, such as Line 317: ‘GGTA1 reads from isolates of 731…’ to ‘GGTA1 reads isolated from 731…’ or ‘ GGTA1 reads from 731…’

Line 366: ‘Inhibition of Cas9 activity was inhibited which was indicated…’ to ‘Inhibition of Cas9 activity was indicated…’

Technical clarifications: Line 409: ‘results were inconclusive.’ Due to? I think the authors may clarify the reason why these results were inconclusive.

Note that off-targets do not imply off-target mutations. Please, the authors should clarify when they refer to off-target sites and when they refer to off-target mutations.

Improve the conclusion to cover their major accomplishments and clarify the lines below as it was a side experiment to search for most likely, but not all, off-target mutations.

Clarify/modify Line 430 – 432: This off-target conclusion seems a premature answer given that their analysis only searched the three most likely off-target sites and does not present a deep unbiased method of genomic search of off-targets, although I agree that Cas9 expressing animals are suitable for in vivo genome editing.
